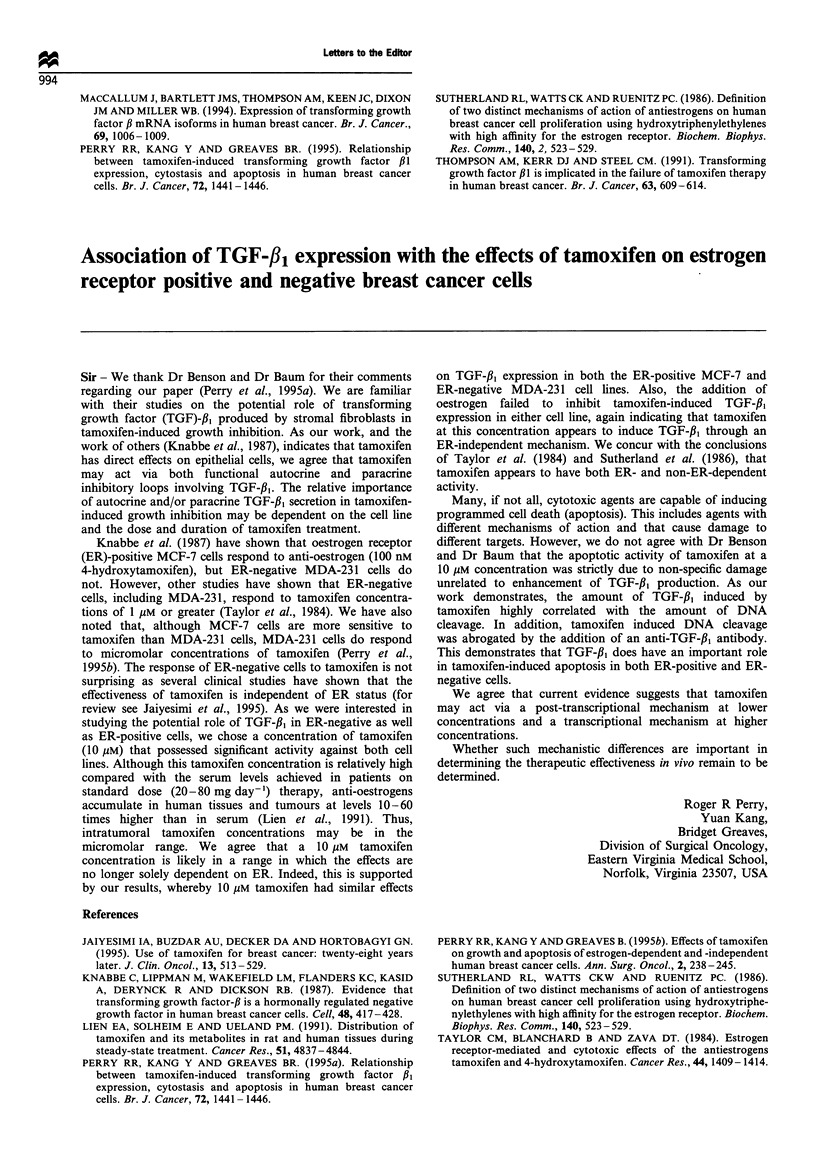# Association of TGF-β_1_ expression with the effects of tamoxifen on estrogen receptor positive and negative breast cancer cells

**Published:** 1996-09

**Authors:** Roger R Perry, Yuan Kang, Bridget Greaves


### Association of TGF-$\beta_1$ expression with the effects of tamoxifen on estrogen receptor positive and negative breast cancer cells

**Sir** – We thank Dr Benson and Dr Baum for their comments regarding our paper (Perry *et al.*, 1995*a*). We are familiar with their studies on the potential role of transforming growth factor (TGF)-$\beta_1$ produced by stromal fibroblasts in tamoxifen-induced growth inhibition. As our work, and the work of others (Knabbe *et al.*, 1987), indicates that tamoxifen has direct effects on epithelial cells, we agree that tamoxifen may act via both functional autocrine and paracrine inhibitory loops involving TGF-$\beta_1$. The relative importance of autocrine and/or paracrine TGF-$\beta_1$ secretion in tamoxifen-induced growth inhibition may be dependent on the cell line and the dose and duration of tamoxifen treatment.

Knabbe *et al.* (1987) have shown that oestrogen receptor (ER)-positive MCF-7 cells respond to anti-oestrogen (100 nM 4-hydroxytamoxifen), but ER-negative MDA-231 cells do not. However, other studies have shown that ER-negative cells, including MDA-231, respond to tamoxifen concentrations of 1 $\mu$M or greater (Taylor *et al.*, 1984). We have also noted that, although MCF-7 cells are more sensitive to tamoxifen than MDA-231 cells, MDA-231 cells do respond to micromolar concentrations of tamoxifen (Perry *et al.*, 1995*b*). The response of ER-negative cells to tamoxifen is not surprising as several clinical studies have shown that the effectiveness of tamoxifen is independent of ER status (for review see Jaiyesimi *et al.*, 1995). As we were interested in studying the potential role of TGF-$\beta_1$ in ER-negative as well as ER-positive cells, we chose a concentration of tamoxifen (10 $\mu$M) that possessed significant activity against both cell lines. Although this tamoxifen concentration is relatively high compared with the serum levels achieved in patients on standard dose (20-80 mg day$^{-1}$) therapy, anti-oestrogens accumulate in human tissues and tumours at levels 10-60 times higher than in serum (Lien *et al.*, 1991). Thus, intratumoral tamoxifen concentrations may be in the micromolar range. We agree that a 10 $\mu$M tamoxifen concentration is likely in a range in which the effects are no longer solely dependent on ER. Indeed, this is supported by our results, whereby 10 $\mu$M tamoxifen had similar effects on TGF-$\beta_1$ expression in both the ER-positive MCF-7 and ER-negative MDA-231 cell lines. Also, the addition of oestrogen failed to inhibit tamoxifen-induced TGF-$\beta_1$ expression in either cell line, again indicating that tamoxifen at this concentration appears to induce TGF-$\beta_1$ through an ER-independent mechanism. We concur with the conclusions of Taylor *et al.* (1984) and Sutherland *et al.* (1986), that tamoxifen appears to have both ER- and non-ER-dependent activity.

Many, if not all, cytotoxic agents are capable of inducing programmed cell death (apoptosis). This includes agents with different mechanisms of action and that cause damage to different targets. However, we do not agree with Dr Benson and Dr Baum that the apoptotic activity of tamoxifen at a 10 $\mu$M concentration was strictly due to non-specific damage unrelated to enhancement of TGF-$\beta_1$ production. As our work demonstrates, the amount of TGF-$\beta_1$ induced by tamoxifen highly correlated with the amount of DNA cleavage. In addition, tamoxifen induced DNA cleavage was abrogated by the addition of an anti-TGF-$\beta_1$ antibody. This demonstrates that TGF-$\beta_1$ does have an important role in tamoxifen-induced apoptosis in both ER-positive and ER-negative cells.

We agree that current evidence suggests that tamoxifen may act via a post-transcriptional mechanism at lower concentrations and a transcriptional mechanism at higher concentrations.

Whether such mechanistic differences are important in determining the therapeutic effectiveness *in vivo* remain to be determined.

Roger R Perry,
Yuan Kang,
Bridget Greaves,
Division of Surgical Oncology,
Eastern Virginia Medical School,
Norfolk, Virginia 23507, USA

### References

JAIYESIMI IA, BUZDAR AU, DECKER DA AND HORTOBAGYI GN. (1995). Use of tamoxifen for breast cancer: twenty-eight years later. *J. Clin. Oncol.*, **13**, 513-529.

KNABBE C, LIPPMAN M, WAKEFIELD LM, FLANDERS KC, KASID A, DERYNCK R AND DICKSON RB. (1987). Evidence that transforming growth factor-$\beta$ is a hormonally regulated negative growth factor in human breast cancer cells. *Cell*, **48**, 417-428.

LIEN EA, SOLHEIM E AND UELAND PM. (1991). Distribution of tamoxifen and its metabolites in rat and human tissues during steady-state treatment. *Cancer Res.*, **51**, 4837-4844.

PERRY RR, KANG Y AND GREAVES BR. (1995*a*). Relationship between tamoxifen-induced transforming growth factor $\beta_1$ expression, cytostasis and apoptosis in human breast cancer cells. *Br. J. Cancer*, **72**, 1441-1446.

PERRY RR, KANG Y AND GREAVES B. (1995*b*). Effects of tamoxifen on growth and apoptosis of estrogen-dependent and -independent human breast cancer cells. *Ann. Surg. Oncol.*, **2**, 238-245.

SUTHERLAND RL, WATTS CKW AND RUENITZ PC. (1986). Definition of two distinct mechanisms of action of antiestrogens on human breast cancer cell proliferation using hydroxytriphenylethylenes with high affinity for the estrogen receptor. *Biochem. Biophys. Res. Comm.*, **140**, 523-529.

TAYLOR CM, BLANCHARD B AND ZAVA DT. (1984). Estrogen receptor-mediated and cytotoxic effects of the antiestrogens tamoxifen and 4-hydroxytamoxifen. *Cancer Res.*, **44**, 1409-1414.